# Around the Hospitals

**Published:** 1892-06-25

**Authors:** 


### Around the Hospitals.

**University College Hospital.** — It must be a matter of regret to the well-wishers of this institution to see the large debt increasing year by year instead of decreasing. So considerable a sum on the wrong side as £14,000 must seriously cripple the efforts of such an institution. A glance at the report of the hospital will show the comprehensive character of the work undertaken. Although a general hospital, University College Hospital includes separate departments for diseases of women and children, the eye, ear, skin, throat, and teeth. The magnitude of the work should find an increasing number of supporters, and we hope next year to learn that legacies, subscriptions, and donations have multiplied, whilst the large deficit is mainly, if not entirely, removed. The hospital is to be closed for six weeks during the summer for extensive repairs, which will represent an additional outlay.

The **West End Hospital for Diseases of the Nervous System, Paralysis, and Epilepsy** now possesses buildings which place it in a condition to compare with any similar institution of the kind in the metropolis. In spite of some difficulties encountered during the year, the number of patients have been maintained and some very successful recoveries have been made. The treatment was originally entirely gratuitous, but an alteration in the rules whereby patients, who are able to do so, are required to pay a small sum, has answered very well. £755 was received as patients' payments during the past year. It is, nevertheless, the intention of the Committee to devote twenty beds entirely free to the indigent poor. It is expected that a further source of income will be derived from classes for instruction in massage and electrical treatment. With the exception of a small deficit on the building fund, the hospital is in a very satisfactory financial position.

The **Longmore Hospital** is the only institution in the South-East of Scotland for incurable patients. So great has been the demand for admission that additions to the building are now being made, and when completed beds for 100 patients will be provided. The fund raised for extension purposes amounts to £10,267, upwards of £4,000 being still required to meet the sum requisite. The rate charged in this institution per patient is £52 per annum in single rooms, and £35 in ordinary wards. It is obvious that these sums cannot defray the actual cost of maintenance, and voluntary contributions are therefore necessary. A slight decrease in these was visible during the past year, but this decrease, it is believed, is only of a temporary nature. Lady collectors render excellent service to the institution, which is greatly indebted to them for a considerable part of its income. The hospital is a branch institution of the Scottish National Society for the Relief of Incurables.

The **North-West London Hospital** has not only been fortunate during the past year in that at the moment of gravest anxiety good friends came to its aid, but also the steadier scource of income, the much desired annual subscription, has increased in number also. Mr. Beckwith Smith's excellent Half-crown Fund is also an additional strength, and altogether we feel that the North-West Hospital has entered into a period of increasing usefulness. The neighbourhood in which the institution is situated especially calls for assistance, there being but small hospital accommodation for a large area populated by the poorest classes. The institution originated through the untiring efforts of two ladies, and commenced its career for out-patients only, and by gradual additions it has arrived at its present size. The list of special beds and cots is most interesting, seeing from whence the maintenance of these is derived. One cot is maintained by the congregation of St. Thomas, another by the Gospel Oak Sunday School, and a third by contributions from the children at St. Paul's Church, Camden Square.

